# X-ray Imaging Versus Anatomical Dissection for Identification of the Fabella

**DOI:** 10.7759/cureus.62186

**Published:** 2024-06-11

**Authors:** Jay M Bauman, Obadah A Alzahabi

**Affiliations:** 1 Center for Anatomical Science and Education, Saint Louis University School of Medicine, Saint Louis, USA

**Keywords:** fabella syndrome, sesamoid, knee, x-ray, fabella

## Abstract

Introduction: Reported prevalence rates of the fabella sesamoid bone vary widely, particularly between studies based on either X-ray imaging or anatomical dissection approaches. The purpose of this study was to directly compare these two methodologies in their detection of fabellae and investigate whether variability in the density of fabellae could explain any discrepancies.

Methods: Fifty cadaveric knee segments were examined for the presence of a fabella by both X-ray imaging and anatomical dissection. The relative density of each excised fabella specimen was then quantified using a separate set of radiographs.

Results: Fabellae were detected in 40% of the sample knees via a manual dissection approach but in just 12% of those same specimens using X-ray imaging. Relative density measurements confirmed that fabellae identifiable only via dissection were significantly less dense than fabellae visible in whole knee radiographs but denser than the surrounding tissue.

Conclusion: Radiology cannot reliably detect cartilaginous or incompletely ossified fabellae, which were found in 28% of the study population. Clinicians should consider the potential occurrence of a fabella when diagnosing posterolateral knee pain, even if it may not be visible via X-ray.

## Introduction

Fabellae are sesamoid bones that develop within the tendon of the lateral head of the gastrocnemius muscle [1,2]. They are most often positioned directly against the posterior side of the lateral condyle of the femur, between which there may be a small synovial cavity [3,4]. Os fabellae have also been observed to possess a variety of tissue types: ossified, cartilaginous, or a mixed composition [2,5,6]. 

Although the role of sesamoid bones in increasing the mechanical advantage of tendons is well established, the function of the fabella itself remains speculative. The traditional interpretation of the fabella function is that of an atavistic structure more useful in quadrupeds, whose knees undergo more rotation and must balance mobility with stability [7,8]. More recent functional speculation has focused on the role of the fabella in reinforcing the soft tissues of the posterolateral corner of the knee, in particular in conjunction with the patellofemoral ligament [2,9].

One reason that the fabella has not received much attention is its perceived lack of clinical importance. “Fabella syndrome” is recognized, which presents most commonly as a complaint of posterolateral knee pain and is often treated by fabellectomy [10-13]. Another symptom associated with fabella syndrome is palsy of the adjacent common fibular nerve [11,14]. Fabellae have also been found to be fractured or dislocated [15,16]. The lack of clinical awareness of the fabella is underscored in case reports as it is often initially misdiagnosed as an osteophyte or intra-articular loose body, or the pain as due to lumbar disc impingement.

The fabellae is also classified as an accessory ossicle due to its variable presence. Curiously, the reported rate of occurrence of fabellae varies dramatically across studies from 3% to 87% [6,17]. One source of variability appears to be the method of identification, as studies based on anatomical dissection reported significantly more fabellae on average (29-32%) than those using X-rays (15-19%) [18,19].

The purpose of the present study was to directly compare X-ray imaging against anatomical dissection as a means of detecting fabellae. We hypothesized that the prevalence rate as assessed by X-ray would be lower than that based on dissection. Additionally, we investigated whether any differences due to the detection methodology could be explained by variability in the degree of fabella ossification. To that end, we hypothesized that any fabellae that are only detectable via anatomical dissection and not X-ray would be less dense than those that are detectable radiologically.

## Materials and methods

Cadaveric knees were obtained following routine dissections performed during a gross anatomy lab course at Saint Louis University (SLU) School of Medicine. Whole knees were isolated by transverse cuts through the thigh and leg segments of the lower limb. Specimens lacking a proximal attachment of the lateral head of gastrocnemius to the surrounding connective tissue of the posterior knee joint capsule were excluded from the study.

Fifty whole-knee specimens were included from a study population consisting of elderly male and female individuals. The age and sex of the donors were not retained after the knee specimens were isolated. All specimens were collected from cadavers received through the SLU Gift Body program in which donors provide written, informed consent. The CASE gift body program abides by all rules set forth by the Uniform Anatomical Gift Act.

**Figure 1:**
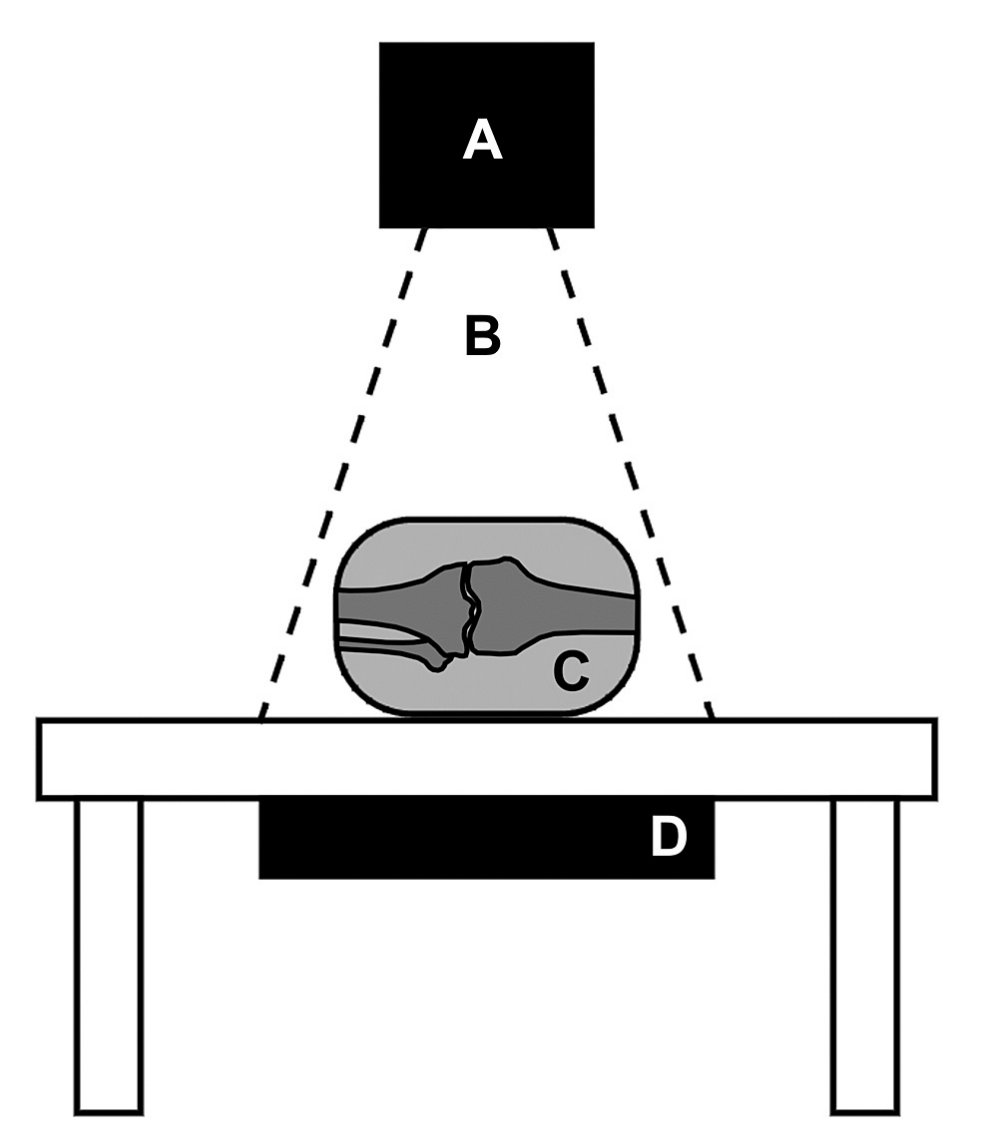
Schematic of X-ray image acquisition setup A: an X-ray generator was mounted over a table. B: an X-ray radiation field was emitted inferiorly from the X-ray generator. C: knee joint specimens were staged such that radiation was projected through the medial or lateral side of the joint. D: a photosensitive plate mounted beneath the table captured the resulting image.

X-ray images were initially collected for each whole knee segment. An X-ray generator (AJEX Meditech, Inc.) mounted above the specimen emitted radiation (70 kv, 2 mAs) through the medial or lateral side of each knee (Figure 1).

**Figure 2:**
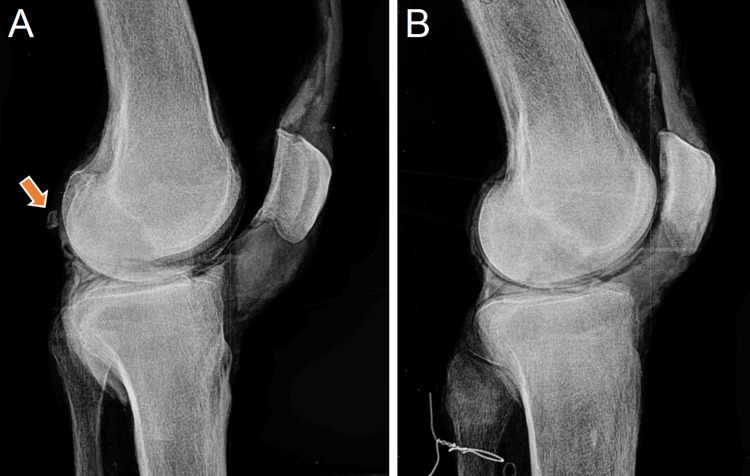
Representative X-ray images used for fabella identification Sagittal plane view of cadaveric knee specimens. A: knee with fabella present (indicated by arrow); B: knee with fabella absent.

The resulting analog image was captured on a photosensitive plate mounted beneath the table, and then subsequently digitized (TigerView software). Representative X-ray images of knee specimens with and without fabellae are presented in Figure 2. All images were checked for alignment of the femoral condyles in the sagittal plane to standardize the perspective across specimens.

**Figure 3:**
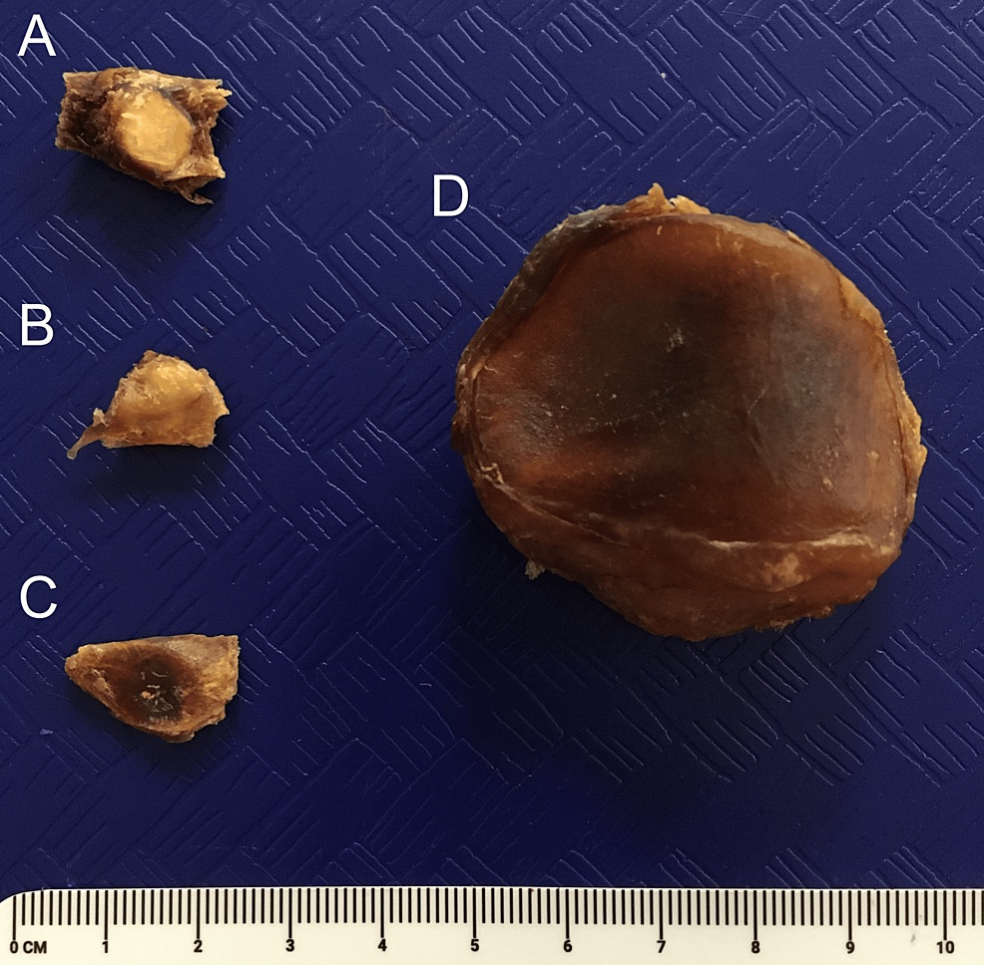
Representative dissected tissue specimens used for fabella identification A: a fabella identifiable via X-ray imaging of the whole knee; B: a fabella identifiable only via anatomical dissection; C: a tissue sample absent of a fabella; D: a patella for reference.

After imaging, each knee joint specimen was dissected to isolate any potential fabellae. The dissection approach was to expose the attachment point of the tendon of the lateral head of the gastrocnemius to the posterior knee joint capsule. During this process, the tendon was manually palpated for the presence of a fabellar mass. Regardless of the outcome of palpation, the tendon and attached joint capsule tissue were removed together and preserved to confirm any negative specimens (i.e., absence of fabella). Finally, the posterior lateral femoral condyle was inspected to verify that none of the samples were the product of an avulsion fracture. Representative tissue specimens obtained from anatomical dissection are presented in Figure 3. All dissections were performed by a single individual to ensure consistency across specimens.

Fabella prevalence among the study population was measured using interrater agreement. Three raters evaluated all 50 X-ray images of the whole knees for the presence or absence of fabellae. The same raters also physically and visually assessed all 50 dissected tissue specimens to determine whether they contained fabellae. A fabella was established to be present based on a standard of two out of three raters agreeing.

**Figure 4:**
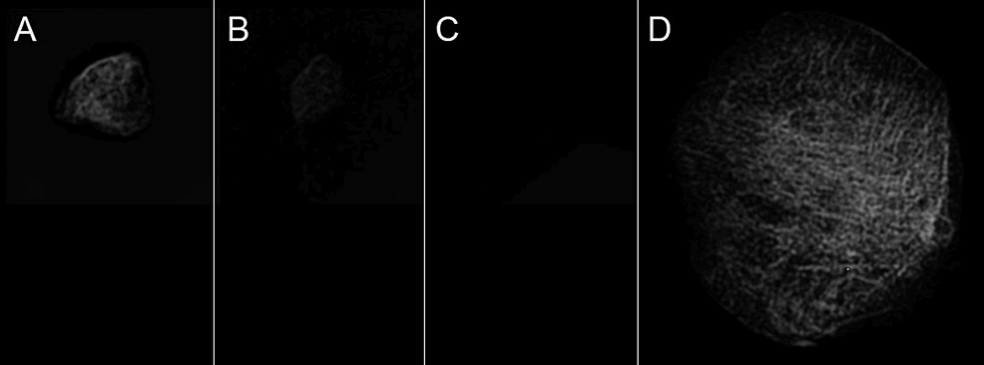
Representative X-ray images of dissected specimens used for relative density calculations A: a fabella identifiable via X-ray imaging of the whole knee (RD = 0.57); B: a fabella identifiable only via anatomical dissection (RD = 0.20); C: a tissue sample absent of a fabella (RD = 0.08); D: a patella for reference (RD = 1).

A new set of X-rays were collected for all 50 dissected tissue samples in order to measure the density of each specimen. The maximum pixel brightness for each individual specimen was recorded (ImageJ software). Pixel brightness values were also measured for tissue specimens that had been assessed as absent of fabellae. To normalize the pixel brightness values across specimens, a patella was included in each image. The patella served as a standard for the absolute maximum brightness value of fully ossified bone. This enabled the calculation of a relative density (RD) value for each specimen by dividing its individual maximum brightness by the absolute maximum brightness of the bony patella. Figure 4 contains representative X-ray images of tissue samples from this portion of the study.

The amount of agreement between raters was quantified using Fleiss’ kappa statistic [20]. Fleiss’ kappa is similar to the intraclass correlation coefficient of ANOVA in that it is a measure of the similarity between groups on a 0-1 scale. Also similarly, these values may be subjectively and qualitatively interpreted, e.g., 0.00-0.20 = poor agreement between groups and 0.21-0.40 = fair agreement.

A Chi-square test was used to compare the prevalence rate between X-ray and dissection. T-tests were used to compare the RD between groups. The level of statistical significance for the Chi-square test and all t-tests was p < 0.05 (SPSS).

## Results

Prevalence rates for each methodology were calculated from the raters’ scores (Table 1). Fabellae were detected in 40% of knees using anatomical dissection compared to 12% using X-ray imaging. This difference between methodologies was significant, X2 (1, N = 50) = 10.19, and p < 0.01.

**Table 1:** Fabella prevalence Fabella prevalence was assessed by both anatomical dissection and X-ray imaging. The interrater agreement (N = 3 raters) for all 50 specimens was also reported.

Detection methodology	Fabella prevalence	Interrater agreement
Dissection	40%	0.81
X-ray	12%	0.24

All fabellae visible via X-ray were encountered in dissection. However, 28% of knees possessed a fabella that was identifiable only via dissection, not X-ray. The interrater agreement score for the anatomical dissection approach was 0.81, which qualifies as an “almost perfect” agreement between raters [20]. However, concordance across raters using X-ray imaging for identification (0.24) was just “fair.”

**Figure 5:**
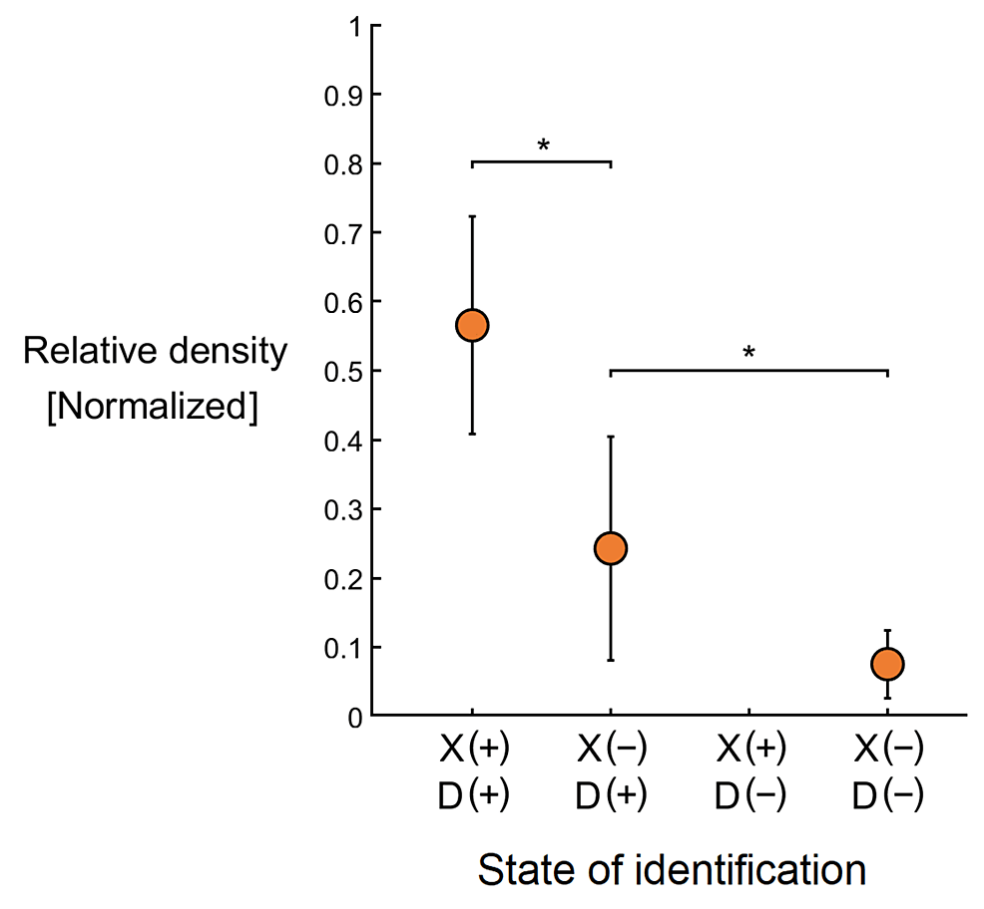
Relative density of fabellae The mean relative density of tissue specimens was grouped by whether each specimen did (+) or did not (-) possess a fabella as assessed by both the X-ray (X) and anatomical dissection (D) methodologies.

Mean relative density values were reported by grouping each tissue specimen based on the methods by which they were detected (Figure 5). The mean density of fabellae visible by both X-ray imaging and anatomical dissection (RD = 0.57 ± 0.16) was significantly greater than that of fabellae only detectable via dissection (RD = 0.24 ± 0.16), p < 0.05. Furthermore, those fabellae only detectable via dissection were significantly denser than tissue samples that were absent of fabellae (RD = 0.08 ± 0.05), p < 0.05.

## Discussion

The difference in fabella prevalence rates (Table 1) based on detection methodology confirms our primary hypothesis that for a given knee specimen, a fabella is significantly less likely to be identified by viewing radiographs (12%) compared to inspecting a dissected tissue specimen (40%). The values in this study are within the ranges reported by systematic reviews of fabella incidence (29-32% for anatomical dissection, 15-19% for X-ray) [18,19]. Additionally, the interrater reliability scores indicate that the identification of fabellae via anatomical dissection is a more consistent approach than looking at radiographs.

The relative density values of dissected fabellae (Figure 4) support our secondary hypothesis that there exists a direct relationship between fabella density and its likelihood of identification via X-ray. The mean RD for each of the three categories of fabella detectability was all significantly different from each other. This supports the interpretation that the discrepancy in prevalence rates between methodologies is largely due to the inability of X-ray to distinguish less ossified fabellae from the surrounding tissues.

Cartilaginous fabellae have been handled in different ways when determining prevalence. Anatomical studies generally classify fabellae into two groups, bony/hard/ossified versus cartilaginous/nonbony/elastic, using manual inspection alone [13,14]. These categorizations have also been confirmed using histological staining [4-6]. Such studies typically include cartilaginous fabellae when calculating prevalence rates.

Radiological studies tend to acknowledge the existence of cartilaginous fabella but do not classify them by tissue type, presumably because any sesamoids identified via X-ray were considered bony [21-27]. However, Zeng et al. found that 8/25 excised fabellae classified as cartilaginous were visible radiologically [4]. Conversely, MRI-based studies are acknowledged to be capable of visualizing cartilaginous fabellae, and, therefore, generally include them in their prevalence rates without classification [26,28,29]. Yet Chew et al., who performed both radiographs and MR imaging of knees, observed no cartilaginous fabellae in their sample [22].

Multiple factors beyond variability in tissue density likely contribute to the heterogeneity in reported fabella prevalence rates. There may be some variation due to ethnicity, as several authors have observed that fabellae seem to be more common in Asian populations [18,19,21,22,24,28]. It’s been speculated that increased ossification in these groups could be driven by lifestyle differences [22]. Fabellae have also been found to be more prevalent in recently performed studies compared to those of past decades [17,19]. There are also methodological inconsistencies between studies, such as whether fabellae are counted per knee or per person [30].

Three demographic factors that would not be expected to contribute to discrepancies in fabella prevalence are the age, sex, and height of the donors. Meta-analyses have demonstrated no significant differences in prevalence rates between males and females and no correlation with either age or height [19].

The direct clinical implication of these findings is that X-ray does not appear to be a reliable method of detecting fabellae when diagnosing posterolateral knee pain. This is important because a fabellectomy does not always eliminate residual pain, and therefore patients should be accurately informed of their surgical and non-surgical therapeutic options [10]. Follow-up studies should investigate whether other imaging methodologies such as MRI and ultrasound are more likely to identify fabellae in a clinical setting. 

Limitations

The knees used in this study lacked information regarding the age, sex, and other demographics of the donors. While having this background would be preferable, literature findings support the conclusion that the absence of age and sex data would not have affected the findings of this study. Additionally, these fabellae were not classified as bony or cartilaginous due to the lack of histological staining.

## Conclusions

This is to our knowledge the first study to directly compare X-ray imaging against anatomical dissection within the same set of subjects for the purpose of fabella identification. Fabellae exhibit a range of densities associated with varying degrees of ossification, which explains the significantly higher prevalence rates based on dissection compared to X-rays. Clinicians should be aware that the fabella is unlikely to be visualized radiologically when diagnosing potential sources of posterolateral knee pain.
